# Supplementary material for: Using ecological propensity score to adjust for missing confounders in small area studies
Source: Biostatistics. 2017 Nov 9;20(1):1–16. doi: 10.1093/biostatistics/kxx058 (PMC6230467; doi:10.1093/biostatistics/kxx058)
Supplement: Supplementary Data [file kxx058_supp.pdf]

Biostatistics (2017), **0**, 0, *pp.* 1–12
doi:10.1093/biostatistics/Appendices

Using Ecological Propensity Score to Adjust for Missing Confounders in Small Area Studies

Yingbo Wang

Novartis, Basel (Switzerland)

Monica Pirani, Anna Hansell

MRC-PHE Centre for Environment and Health, Imperial College London (UK)

Sylvia Richardson

MRC Biostatistics Unit, Cambridge University (UK)

Marta Blangiardo*

MRC-PHE Centre for Environment and Health, Imperial College London (UK)

m.blangiardo@imperial.ac.uk

∗ To whom correspondence should be addressed.

1. Multivariate CAR

The BYM model proposed by Besag et al. (1991) is assigned to $\psi_{1qd} + \psi_{2qd}$ for district d:

$$\psi_{1qd}|(\psi_{1ql,\ l\neq d}) \sim \text{Normal}\left(\frac{\sum_{l\in\mathcal{N}(d)}\psi_{1ql}}{\mathcal{N}(d)}, \frac{\sigma^2_{\psi_{1q}}}{\mathcal{N}(d)}\right) \tag{1.1}$$

$$\psi_{2qd} \sim \text{Normal}(0, \sigma^2_{\psi_{2q}})$$

where ψ_{1qd} is specified through the intrinsic conditional autoregressive model (iCAR) proposed by Besag and Kooperberg (1995), while ψ_{2qd} follows a normal distribution with a common variance $\sigma^2_{\psi_{2q}}$. For the qth confounder, a local smoothing is provided by ψ_{1qd} based on the values of the set of neighbours $\mathcal{N}(d)$ and a global smoothing is included through ψ_{2qd} based on the values of all the

© The Author 2017. Published by Oxford University Press. All rights reserved. For permissions, please e-mail: journals.permissions@oup.com

units. Note that the iCAR is improper, as it is possible to add a constant to each ψ_{1qd} without changing the distribution, so a global intercept α_q needs to be added as well as a sum-to-zero constraint. We followed the specification provided in Lunn et al. (2012) pag 264 and on α_q we specified a flat distribution between $\pm\infty$, leading to the joint prior of the intercept and random effects to be equivalent to specify an iCAR prior on the random effects without constraint.

A correlation structure between the confounders should be included to allow borrowing of strength, as some might have been collected on several years, thus being available for many individuals, while other might not. We extend $\sigma^2_{\psi_{1q}}$ in (1.1) to Σ_{ψ_1} and equivalently $\sigma^2_{\psi_{2q}}$ to Σ_{ψ_2}; the diagonals model the variances for each confounder (spatially structured and non), while the off diagonal identifies the covariances among confounders. This specification leads to the multivariate BYM model (MVBYM). See Gamerman et al. (2003), Thomas et al. (2004) for details and applications of MVBYM.

2. The specification of RW(2)

We choose a T-state RW(2) as $f()$ in the imputation model. First, each continuous variable $\boldsymbol{C}_p$ from (2.3) in the main text is converted into a categorical variable by cutting its space into T equally spaced states ($t = 1, \dots, T$), so that each C_{ip} becomes $C_{cat(t_i)p}$. Then the non-linear relationship between $\boldsymbol{C}_p$ and EPS is approximated by $C_{cat(t_i)p}$ and s_t where s_t is the value estimated by RW(2) on the tth state, i.e. $f(\mathrm{C}_{cat(t_i)p})$; RW(2) links 1st and 2nd order neighbours of s_t through the following conditional distribution:

$$s_t|s_{-t} \sim \begin{cases} \text{Normal}(2s_{t+1} - s_{t+2}, \sigma^2) & \text{for} \quad t = 1 \\ \text{Normal}(\frac{2}{5}s_{t-1} + \frac{4}{5}s_{t+1} - \frac{1}{5}s_{t+2}, \frac{1}{5}\sigma^2) & \text{for} \quad t = 2 \\ \text{Normal}(-\frac{1}{6}s_{t-2} + \frac{2}{3}s_{t-1} + \frac{2}{3}s_{t+1} - \frac{1}{6}s_{t+2}, \frac{1}{6}\sigma^2) & \text{for} \quad t = 3, ..., T-2 \\ \text{Normal}(-\frac{1}{5}s_{t-2} + \frac{4}{5}s_{t-1} + \frac{2}{5}s_{t+1}, \frac{1}{5}\sigma^2) & \text{for} \quad t = T-1 \\ \text{Normal}(-s_{t-2} + 2s_{t-1}, \sigma^2) & \text{for} \quad t = T \end{cases} \tag{2.2}$$

The same specification is considered for the link between EPS and λ in the analysis model (Equation 2.4 in the main text).

3. The simulation process

The data simulation process is described below:

1. Simulate one set of ecological variables $X, \boldsymbol{C} = (\boldsymbol{C}_1, \boldsymbol{C}_2), \boldsymbol{M} = (\boldsymbol{M}_1, \boldsymbol{M}_2, \boldsymbol{M}_3, \boldsymbol{M}_4)$. Let i denote the area index with $i = 1, \ldots, 300$ ($i \in S \cup I$). Simulate $\boldsymbol{C}$ based on the expit transformation of Normal$(0,1)$, and generate correlated $\boldsymbol{M}$ based on the expit transformation of bivariate normal distribution:

$$\begin{pmatrix} \text{logit}(\text{M}_{1i}) \\ \text{logit}(\text{M}_{2i}) \end{pmatrix} \sim \text{MVN}_2\left(\begin{bmatrix} 0 \\ 0 \end{bmatrix}, \sigma^2 \begin{bmatrix} 1 & 0.3 \\ 0.3 & 1 \end{bmatrix}\right)$$
$$\begin{pmatrix} \text{logit}(\text{M}_{3i}) \\ \text{logit}(\text{M}_{4i}) \end{pmatrix} \sim \text{MVN}_2\left(\begin{bmatrix} 0 \\ 0 \end{bmatrix}, \sigma^2 \begin{bmatrix} 1 & 0.3 \\ 0.3 & 1 \end{bmatrix}\right)$$

where $\sigma = 1$. Then the exposure X is produced through a Bernoulli distribution:

$$X_i \sim \text{Bernoulli}(P(X_i = 1|\boldsymbol{C}_i, \boldsymbol{M}_i))$$
$$\text{logit}(P(X_i = 1|\boldsymbol{C}_i, \boldsymbol{M}_i)) = \theta_1 + \boldsymbol{C}_i^T \boldsymbol{\theta}_C + \boldsymbol{M}_i^T \boldsymbol{\theta}_M$$
$$\text{EPS}_i = \boldsymbol{M}_i^T \boldsymbol{\theta}_M$$

where $\boldsymbol{\theta}_C = (\theta_2, \theta_3)$ and $\boldsymbol{\theta}_M = (\theta_4, \theta_5, \theta_6, \theta_7)$. The true values for $\boldsymbol{\theta}$ are set to be the following:

$$\theta_1 = 0; \theta_2 = 0.5; \theta_3 = -0.5; \theta_4 = 1; \theta_5 = -0.6; \theta_6 = 0.5; \theta_7 = -0.4$$

Suppose the expected count E is the same across all areas, i.e. $E_i = 100 \ \forall \ i = 1, \ldots, 300$, then the observed count O is simulated through:

$$O_i \sim \text{Poisson}(E_i \lambda_i)$$
$$\log(\lambda_i) = \beta_1 + \beta_2 X_i + \boldsymbol{C}_i^T \boldsymbol{\beta}_C + \boldsymbol{M}_i^T \boldsymbol{\beta}_M \qquad (3.3)$$

The true values for β_1, β_2, $\boldsymbol{\beta}_C = (\beta_3, \beta_4)$ and $\boldsymbol{\beta}_M = (\beta_5, \beta_6, \beta_7, \beta_8)$ are:

$$\beta_1 = 0; \beta_2 = 0.5 \text{ or } 0; \beta_3 = 0.2; \beta_4 = -0.2; \beta_5 = 0.2; \beta_6 = -0.2; \beta_7 = 0.2; \beta_8 = -0.2$$

2. Simulate n values for the individual variables $\boldsymbol{m} = (\boldsymbol{m}_1, \boldsymbol{m}_2, \boldsymbol{m}_3, \boldsymbol{m}_4)$:

$$m_{qij} \sim \text{Bernoulli}(M_{qi}), q = 1, ..., 4,\ j = 1, ..., n$$

   where n is chosen to be 5, 10, 20 and 100 for the different simulation scenarios.

3. The following MAR criterion is then applied to remove $\boldsymbol{M}$ or $\boldsymbol{m}$ from around 50% of the areas:

$$l_i = \text{Bernoulli}(P(l_i = 1)) \tag{3.4}$$
$$\text{logit}(P(l_i = 1)) = 0.2C_{1i} - 0.2C_{2i}$$

   where l_i is the indicator for the missingness of $\boldsymbol{M}_i$ or $\boldsymbol{m}_i$. The complete cases are defined as the areas with $\boldsymbol{M}_i$ or $\boldsymbol{m}_i$ available ($l_i = 0$, i.e. $i \in S$), while the remaining areas have missing $\boldsymbol{M}_i$ or $\boldsymbol{m}_i$, i.e. $i \in I$.

The simulation result with true $\beta_2 = 0$ is shown in Table 1.

Table 1: EPS performance and comparison with MICE on the simulation study (true $\beta_2 = 0$, 100 simulated datasets).

	Adjustment/ Imputation	Posterior Mean for β_2	Bias	RMSE	CI95 width	CI95 coverage
**Scenario 1: *M* are available in all areas**						
	Direct adj	0.00	0.00	0.02	0.060	0.94
	EPS adj	0.00	0.00	0.02	0.064	0.93
**Naïve case: Ignoring *M***						
	NA	0.28	0.28	0.28	0.054	0.00
**Scenario 2: *M* are only available in some areas**						
Case 2.1: Analysis on $i \in S$	EPS adj	0.00	0.00	0.03	0.093	0.91
Case 2.2 : Analysis on	MICE	0.03	0.03	0.04	0.104	0.81
$i \in S \cup I$	EPS imput	-0.01	-0.01	0.03	0.088	0.89
**Scenario 3: *M* are NOT directly available, but *m* are available in some areas**						
**Sample size n=5**						
Case 3.1.1: Analysis on $i \in S$	EPS adj	0.09	0.09	0.11	0.087	0.24
Case 3.2.1: Analysis on	MICE	0.13	0.13	0.14	0.142	0.18
$i \in S \cup I$	EPS imput	0.08	0.08	0.08	0.092	0.31
**Sample size n=10**						
Case 3.1.2: Analysis on $i \in S$	EPS adj	0.09	0.09	0.11	0.088	0.40
Case 3.2.2: Analysis on	MICE	0.13	0.13	0.13	0.139	0.45
$i \in S \cup I$	EPS imput	0.08	0.08	0.08	0.091	0.56
**Sample size n=20**						
Case 3.1.3: Analysis on $i \in S$	EPS adj	0.08	0.08	0.10	0.085	0.54
Case 3.2.3: Analysis on	MICE	0.10	0.10	0.11	0.139	0.50
$i \in S \cup I$	EPS imput	0.07	0.07	0.08	0.089	0.62
**Sample size n=100**						
Case 3.1.4: Analysis on $i \in S$	EPS adj	0.02	0.02	0.05	0.091	0.74
Case 3.2.4: Analysis on	MICE	0.06	0.06	0.06	0.126	0.61
$i \in S \cup I$	EPS imput	0.01	0.01	0.04	0.089	0.83

4. Simulation study to compare EPS imputation specification

There are two ways to include the information of X in the imputation model:

1. including X as a predictor (PredX):

$$\text{EPS}_i = \eta_1 + \gamma X_i + f(,\boldsymbol{C}_i) + \phi_{d_i} \tag{4.5}$$

2. including X as a response variable (RespX):

$$\text{logit}(P(X_i = 1|\boldsymbol{C}_i, \text{EPS}_i)) = \theta_1 + \boldsymbol{C}_i^T \boldsymbol{\theta}_C + \text{EPS}_i \tag{4.6}$$

$$\text{EPS}_i = \eta_1 + f(\boldsymbol{C}_i) + \phi_{d_i}$$

The former (PredX) is a standard way of including covariates in the imputation model (Kenward and Carpenter, 2007), whereas the latter (RespX) is used by McCandless et al. (2012) to define the relationship between EPS_i and X. RespX is natural for EPS imputation since it follows the EPS estimation model, but the specification of RespX requires an additional logistic regression (4.6), which is computationally less efficient than PredX.

4.1 *The simulation to compare RespX and PredX*

It seems little research has been done on the comparison of these two different specifications involving exposure X in modelling missing EPS, and so the following simulation study is designed to compare the performance of PredX and RespX.

4.1.1 *Simulation design* The simulation uses the datasets generated from Section 3 with the true imputation model specified as $\text{EPS}_i = -3 + |C_{1i}| + |C_{2i}| + \text{Normal}(0, 0.5)$ and with true $\mathbf{Y}$ generated from $Y_i = X_i + C_{1i} - C_{2i} + 0.2(\text{EPS}_i + 2)^2 + \text{Normal}(0, 2)$. The following missing probability model is specified to create around 50% of the missing EPS: $\text{logit}(P(\text{EPS}_i = NA|X_i, \boldsymbol{C}_i, Y_i)) = 0.2C_{1i} - 0.2C_{2i}$. Each scenario contains 100 datasets, and four statistics (mean, RMSE, CI coverage and CI width at 95% level) are computed for the estimated β_2.

The simulation initially assesses whether X should be included in the imputation process in the case where the imputation function $f()$ linking confounders $\boldsymbol{C}$ is either truly specified or

ignored. Then it compares two specifications of X in the imputation model: PredX (treat X as a predictor) and RespX (treat X as a response variable). To focus on the comparison of PredX and RespX, the true link function $h() = 0.2(\text{EPS}_i + 2)^2$ is used.

4.1.2 *Simulation results* The simulation results are shown in Table 2. The simulation shows that it is important to include the information of X in the imputation process, either through PredX or RespX, especially in the case where the imputation function $f()$ is not correctly specified (in reality, the imputation function $f()$ is indeed unknown). For example, the first three rows show that the bias of β_2 is reduced from 0.84 to a negligible value by including the information of X. The simulation also suggests that the format of including the information X is not critical due to the largely indistinguishable results between PredX and RespX.

Table 2: The comparison of the specification of PredX and RespX based on the estimation of β_2 on 100 simulated datasets

Link func. g()	Imputation model	True value of β_2	Posterior Mean	Bias	RMSE	CI95 coverage	CI95 width
True h()	Ignore. $f(\boldsymbol{C})$	1	1.84	0.84	0.88	0.18	1.20
True h()	Ignore. $f(\boldsymbol{C})$ + PredX	1	0.98	-0.02	0.30	0.95	1.24
True h()	Ignore. $f(\boldsymbol{C})$ + RespX	1	1.04	0.04	0.28	0.95	1.21
True h()	True $f(\boldsymbol{C})$	1	1.04	0.04	0.28	0.98	1.10
True h()	True $f(\boldsymbol{C})$ + PredX	1	0.95	-0.05	0.29	0.96	1.11
True h()	True $f(\boldsymbol{C})$ + RespX	1	0.95	-0.05	0.28	0.96	1.12

5. Simulation study designed to assess the model performance in presence of spatial structure and non-linearity

We designed and run an additional simulation study to assess the performance of our model in presence of spatial structure and non-linear relationship between unmeasured confounding factors and the health outcome. The synthetic data are generated broadly following the main simulation design presented in Section 3, with few exceptions. In particular, in the interest of simplicity, we

assume only one ecological confounder C and to allow for spatial structure and non-linearity we assume that two of the four unmeasured ecological confounders are continuous instead of binary. We simulate 100 replicated data sets as follows:

1. Simulate a spatial region comprising 300 areas on a regular 20×15 grid and construct a binary 300×300 spatial neighbourhood matrix for this region based on spatial adjacency. Generate a spatial random effect $\boldsymbol{\varphi}$ using a Gaussian process representation with zero mean and covariance function described by an exponential model (e.g. Banerjee et al., 2014). Simulate a high-order 4×5 super-grid that incorporates the original grid cells in cluster of 15 cells. This mimic the real-world application presented in the main paper, where larger spatial units (i.e. local authority districts) are used for modelling spatially structured random effects in the EPS estimation and imputation to overcome the issue of spatial sparsity at ward level.

2. Simulate a set of ecological variables $X, C, \boldsymbol{M}_i = (M_{i1}, \ldots, M_{i4})$ for each area $i = 1, \ldots, 300$ ($i \in S \cup I$). In particular, simulate (i) C from an independent standard normal, (ii) $M_{i1:2}$ from the expit transformation of a bivariate normal distribution (as specified in Section 3 of Supplementary Material), (iii) M_{i3} from a Normal$(\varphi_i, 0.3^2)$, and (iv) M_{i4} from Normal$(0, 0.3^2)$. Then, simulate the exposure X from a Bernoulli with parameter distribution depending from C and $\boldsymbol{M}_i$ (similarly to Section 3).

3. Fit a non-linear sine curve to M_{i4}, such that $s(M_{i4}) = \sin(3\pi M_{i4}) \exp(-M_{i4})$ and simulate the health response O assuming a Poisson likelihood with parameter of the distribution being a log-linear function of X, C, $M_{i1:3}$ and $s(M_{i4})$. In this way, the model is still a linear functions of the parameters, but is non-linear with respect to the input variable M_{i4}. Here, the inclusion of a random effect exhibiting spatial structure is not needed as M_{i3} induces spatial autocorrelation into the health response. The Moran's I (Moran, 1950), that is a

global test of autocorrelation, confirms that the response O hold a spatial structure (p-value < 0.01 for all the data sets).

4. For each area i, simulate $n = 20$ values for the individual variables $\boldsymbol{m}_{qi} = (m_{qi1}, \ldots, m_{qin})$. In particular, simulate $m_{1:2ij}$ from a Bernoulli distribution based on the proportion of $M_{1:2i}$ and generate $m_{3:4ij}$ from a normal distribution with location parameter derived from $M_{3:4i}$.

5. Then, use a MAR mechanism (depending on C) to remove $\boldsymbol{m}_{qi}$ from around 50% of the areas, according a binary missing value indicator: $l_i \sim \text{Bernoulli}(p_i)$ with $\text{logit}(p_i) = 0.2C_i$.

In this simulation study, we keep the set of the true parameter values as described in Section 3 (note that for the ecological confounder C we use the coefficients used in Section 3 for C_1).

The analyses performed for the EPS estimation, imputation and adjustment follow the modelling approach described in Sections 2 of the main paper. Table 3 presents the results of this simulation study, assuming the true value of $\beta_2 = 0.5$. Using direct adjustment we can appreciate that if the non-linearity in the relationship between M_4 and Y is ignored the RMSE increases and the coverage decreases. At the same time scenario 2 shows that there is bias of 0.11 and a RMSE of 0.12 when only C and X are included in the analyses. Scenario 3 shows that the EPS framework returns unbiased results; the root mean square error increases slightly with respect to the direct adjustment, but the coverage increases to 72% when the spatial structure is also considered in the model from 62% seen for the direct adjustment when M_4 is included as linear.

Table 3: Performance of the EPS framework when spatial structure and non-linear relationship between unmeasured confounding variables and health outcome are present.

Adjustment	Posterior Mean for β_2	Bias	RMSE	CI95 width	CI95 coverage
**Scenario 1: $\boldsymbol{M}$ are available in all areas**					
Direct adj ($M_{i1:4}$ included in the health model as linear)	0.50	0.00	0.03	0.046	0.62
Direct adj ($M_{i1:3}$ included in the health model as linear, M_{i4} as Fourier sine transformed)	0.50	0.00	0.02	0.045	0.96
**Scenario 2 (Näive case): Ignoring $\boldsymbol{M}_i$**					
NA	0.61	0.11	0.12	0.041	0.00
**Scenario 3: $\boldsymbol{M}_i$ are NOT directly available, but $\boldsymbol{m}_{ij}$ are available in some areas**					
EPS adj accounting for the spatial structure in the imputation model	0.50	0.00	0.04	0.059	0.72
EPS adj ignoring the spatial structure in the imputation model	0.50	0.00	0.04	0.062	0.67

6. Air pollution and Health in Greater London

6.1 *Convergence*

The following figure shows the potential scale reduction factor ($\hat{R}$); it represents how the credible intervals would be reduced if the MCMC simulation were to run forever. It is recommended as a measure of parameter convergence (Gelman and Hill, 2006) and the usual practice is to continue the simulation until $\hat{R} < 1.1$. We can see that for our analysis $\hat{R}$ is always smaller than 1.1, suggesting good convergence.

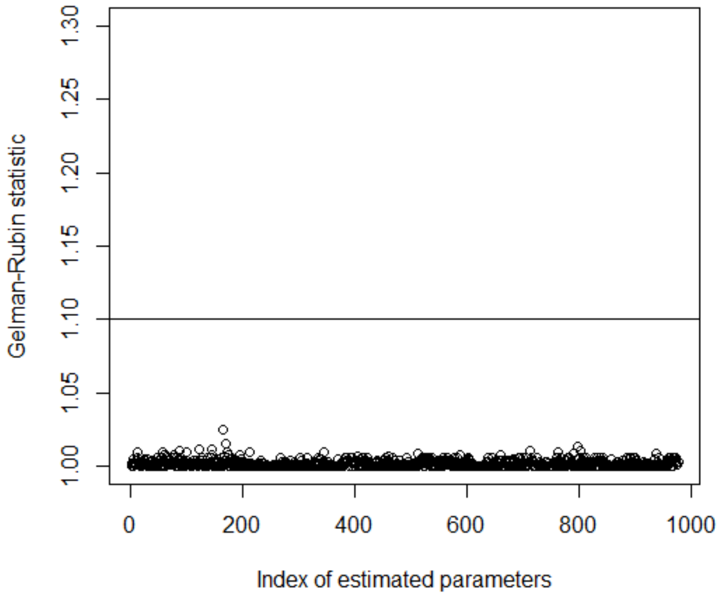

Figure 1: $\hat{R}$ for all the parameters estimated through our proposed modelling framework in the illustrative example.

6.2 *Changing the exposure threshold*

Table 4: Relative risk (RR) of hospital admission: PM_{10} threshold equal to $24\mu g/m^3$ or $26\mu g/m^3$ (25% or 75% of the PM_{10} distribution) in Greater London. The table shows that the results are consistent with what we obtain using a 50% threshold on the distribution of PM_{10}.

Areas	Data used	$24\mu g/m^3$ threshold ($\geqslant$ 5 subjects)		$26\mu g/m^3$ threshold $\geqslant$ 5 subjects	
		RR (CI95)	CI95 width	RR (CI95)	CI95 width
$i \in S$	X,**C**	0.95 (0.88-0.99)	0.11	0.94 (0.89-1.00)	0.11
	X,**C**,EPS	1.05 (0.97-1.13)	0.16	1.08 (1.01-1.15)	0.14
$i \in S \cup I$	X,**C**	0.94 (0.89-1.00)	0.11	0.88 (0.83-0.93)	0.10
	X,**C**,EPS	1.02 (0.95-1.09)	0.14	1.03 (0.96-1.09)	0.13
	X,**C**,MICE	0.94 (0.90-0.99)	0.10	0.91 (0.85-0.97)	0.12

References

Banerjee, S., Gelfand, A. E., and Carlin, B. P. (2014). *Hierarchical Modeling and Analysis for Spatial Data*. Chapman & Hall/CRC, Boca Raton, FL, 2nd edition.

Besag, J. and Kooperberg, C. (1995). On conditional and intrinsic autoregressions. *Biometrika*, 82(4):733–746.

Besag, J., York, J., and Molli'e, A. (1991). Bayesian image restoration, with two applications in spatial statistics. *Annals of the Institute of Statistical Mathematics*, 43(1):1–20.

Gamerman, D., Moreira, A. R. B., and Rue, H. (2003). Space-varying regression models: specifications and simulation. *Computational Statistics & Data Analysis*, 42(3):513–533.

Gelman, A. and Hill, J. (2006). *Data analysis using regression and multilevel/hierarchical modelling*. Cambridge University Press.

Kenward, M. G. and Carpenter, J. (2007). Multiple imputation: current perspectives. *Statistical Methods in Medical Research*, 16(3):199–218.

Lunn, D., Jackson, C., Best, N., Thomas, A., and Spiegelhalter, D. (2012). *The BUGS Book: A Practical Introduction to Bayesian Analysis*. CRC Press.

McCandless, L. C., Richardson, S., and Best, N. (2012). Adjustment for Missing Confounders Using External Validation Data and Propensity Scores. *Journal of the American Statistical Association*, 107(497):40–51.

Moran, P. A. P. (1950). Notes on continuous stochastic phenomena. *Biometrika*, 31:17–23.

Thomas, A., Best, N., Lunn, D., Arnold, R., and Spiegelhalter, D. (2004). *GeoBUGS User Manual V1.2*. MRC Biostatistical Unit.
